# Trend, Epidemiology, and Clinical Characteristics of Vulvar Cancers in Lagos, Nigeria: A Facility-Based Study

**DOI:** 10.7759/cureus.83041

**Published:** 2025-04-26

**Authors:** Adeyemi A Okunowo, Oluwatoyin M Olayioye, Muhammad Y Habeebu, Chinedu C Anumni, Ephraim O Ohazurike, Kehinde S Okunade, Rose I Anorlu

**Affiliations:** 1 Obstetrics and Gynaecology, College of Medicine, University of Lagos/Lagos University Teaching Hospital, Lagos, NGA; 2 Obstetrics and Gynaecology, Lagos University Teaching Hospital, Lagos, NGA; 3 Radiation Oncology, College of Medicine, University of Lagos/Nigeria Sovereign Investment Authority-Lagos University Teaching Hospital (NSIA-LUTH), Lagos, NGA

**Keywords:** clinical characteristics, epidemiology, gynaecological cancers, lagos nigeria, trend, vulvar cancer

## Abstract

Background

Vulvar cancer (VC) is a rare gynecological cancer (GC) that is not commonly studied. Because of its location, vulvar symptoms are not frequently brought to the clinician’s attention. Many women are not aware of VC and frequently attribute its early symptoms to other benign causes. In addition, little is known about the disease epidemiology and clinical characteristics in Lagos, Nigeria.

Objectives

Our study aimed to describe the trend, epidemiology, and clinical characteristics of VC in Lagos, Nigeria.

Materials and methods

Records of women with VC who presented to Lagos University Teaching Hospital between January 2010 and December 2019 were retrieved, and information on their socio-demographic and clinical characteristics was obtained for analysis. Data analysis was done using IBM Corp. Released 2017. IBM SPSS Statistics for Windows, Version 25.0. Armonk, NY: IBM Corp.

Results

VC accounted for 4.0% of all GC managed in the hospital. There was a rising trend in the number of VCs managed during the study period, with 75.9% of cases managed in the last five years. The mean ± SD age of women with VC was 52.2 ± 11.8 years, with 27 (60.0%) above 50 years old. The majority had low-level occupations, 35 (77.8%), multiple sexual partners, 29 (64.4%); history of genital warts, 35 (77.8%); vulvar skin lesions, 40 (88.9%), and were menopausal, 26 (57.8%). The most common presenting symptom was vulvar swelling 32 (71.1%) and 20 (44.4%) presented with stage III disease. Squamous cell carcinoma was the most common histological type, 35 (77.8%), while the use of only surgery, 15 (33.3%), chemotherapy, 4 (8.9%), radiotherapy, 4 (8.9%), and chemoradiation, 4 (8.9%), were the most common treatment modalities.

Conclusion

VC accounts for 4.0% of GC in Lagos, Nigeria, and its prevalence is rising. Women with VC are usually advanced in age, postmenopausal, have a history of multiple sexual partners, and frequently present in advanced-stage disease. There is a need to increase awareness about the disease and support its care to improve health outcomes.

## Introduction

The vulva is an essential part of the female genitalia called the external genitalia, and it comprises the labia majora, labia minora, clitoris, bulb of the vaginal vestibule, the lesser vestibular glands also called the Skene glands, and the greater vestibular glands known as the Bartholin’s glands [1]. It is a private region of the female’s body reserved for sexual and reproductive activities, and cancer can arise from any of its components. Vulva cancer (VC) is a rare gynecological malignancy, accounting for barely 1% of all female cancers and 4% of all reproductive tract cancers [1,2]. According to the GLOBOCAN 2022 report, VC is the fourth most common genital tract cancer, after cervical, uterine, and ovarian cancers, with 47,342 new cases and 18,579 cancer deaths reported worldwide, accounting for 0.2% of the total new cases of cancers and cancer deaths, respectively, in 2022 [3]. The incidence and mortality burden of VC is highest in Europe and Asia, with Africa accounting for an incidence and mortality rate of 11.9% and 16.1%, respectively [4]. In Nigeria, 1056 new cases and 579 deaths were attributed to VC in 2022, accounting for less than 1% of all new cancer cases and cancer-related deaths, respectively [5].

Approximately 80-95% of VC are squamous cell carcinoma (SCC) in origin, followed by melanoma and other rare histologic types [1,6]. There are two major histological variants of SCC, and they arise from different etio-pathological pathways with distinct characteristics. Keratinizing SCCs are human papillomavirus (HPV)-independent, arising from chronic vulvar dystrophies like lichen sclerosus, squamous hyperplasia, and differentiated vulvar intraepithelial neoplasia (dVIN). This is the most common variant, accounting for about 60% of all SCC, and it typically occurs in older women above 60 years. Warty or basaloid SCC is an HPV-associated cancer, commonly caused by high-risk HPV (hr-HPV) similar to the ones causing cervical cancer, especially HPV 16, 18, 31, and 33. It is usually multifocal in origin, occurs in younger women, and accounts for approximately 30% of all SCC [1,6]. SCC may arise from preneoplastic lesions in the vulvar and anal regions generally referred to as lower anogenital squamous intraepithelial lesions. Low squamous intraepithelial lesions (LSIL), formerly referred to as vulvar intraepithelial neoplasia 1 (VIN 1), and high squamous intraepithelial lesions (HSIL), formerly referred to as VIN 2 & 3, are precursor lesions of HPV-associated SCC, while dVIN is associated with chronic vulvar dermatoses and keratinizing SCC with more aggressive malignant behavior compared with HSIL [1,6].

Due to its occurrence at a late age, VC is typically referred to as a disease of menopause. However, following the advent of HIV/AIDS and the high prevalence of HPV infection, its incidence has significantly increased, and the age at onset of the disease has decreased [1,6]. The risk factors for VC are multifaceted and include age, HR-HPV infections, HIV/AIDS, history of cervical cancer, presence of cervical or vulvar HSIL, chronic vulvar dystrophies such as lichen sclerosus, smoking, and immunosuppression [7]. Bucchi et al. [8], in a systematic review, reported on other risk factors associated with VC, such as the presence of other sexually transmitted diseases, autoimmune diseases such as systemic lupus erythematosus, familial clustering of HPV-associated cancers, metabolic syndrome, diabetes mellitus, and high body mass index. Similarly, sexual behavior and practices like early age at coitarche, high number of sexual partners, type of sexual intercourse, total number of marriages, and age at first marriage have been reported to be associated with the risk of VC, though with some conflicting results [8]. Women with VC can either present with symptoms or be asymptomatic. Common symptoms include pruritus vulvae, vulvar growth or nodule, ulcerative lesions, vulvar pain, discharge, bleeding, or dysuria [9,10].

VC has been poorly studied worldwide due to the rarity of the disease, leading to a lack of adequate statistical data to guide recommendations and management [11,12]. Studies on VC are very few in Nigeria, resulting in a poor understanding of the epidemiology, clinical characteristics, and management strategy of the disease among Nigerian women. Among the few studies conducted in Nigeria [13-16], none has been conducted in Lagos State, Nigeria, leaving a huge knowledge gap in the understanding of the epidemiology and characteristics of VC in a cosmopolitan and the most populous city in Nigeria and Africa. To bridge this gap, our study aimed to examine the trend of VC and describe the epidemiology and clinical characteristics of the disease in Lagos State, Nigeria.

## Materials and methods

Study design and setting 

This was a retrospective cross-sectional study conducted among women with histological diagnoses of VC who received care at the Lagos University Teaching Hospital (LUTH), Lagos State, Nigeria. LUTH is the main and largest referral center for cancer management in Lagos State, southwestern Nigeria. It has approximately 760 beds and caters to the huge population of about 25 million people living in the twenty local government areas of the state [17] and its neighborhood. It has facilities for complex cancer surgery, radiation therapy, chemotherapy, and palliative care.

Study population and eligibility criteria

This included all women with histological diagnoses of VC managed at LUTH over a 10-year period from January 1st, 2010, to December 31st, 2019. Women who had histologically confirmed VC during the study period with complete data were enrolled in the study, while women without histological confirmation of VC or with missing or incomplete data were excluded from the study.

Data collection

The medical record register of all patients who attended the LUTH gynecological oncology outpatient clinic, gynecological accident & emergency unit, and radiotherapy outpatient clinic or were admitted into the female in-lying wards during the 10-year study period was reviewed to identify women with a diagnosis of VC who received care in the hospital during the period. The medical records of these women were identified and retrieved from the medical records department, and appropriate information was obtained using a structured study proforma. Information on sociodemographic characteristics, reproductive characteristics, risk factors for VC, and symptoms at presentation was obtained. Information on the stage at presentation, histological types, and mode of treatment of VC were also retrieved from the case notes. The information on the number of women managed for gynecological malignancies in the institution during the study period was also obtained to determine the burden of VC relative to other gynecological malignancies.

Data analysis

The collected data was entered into a Microsoft Excel spreadsheet, de-identified, cleaned, and validated. Statistical analysis was done using IBM Corp. Released 2017. IBM SPSS Statistics for Windows, Version 25.0. Armonk, NY: IBM Corp. Variables were grouped into continuous and categorical variables. Continuous variables were tested using the Shapiro-Wilk test for normal distribution, and normally distributed and skewed variables were expressed as mean ± standard deviation (SD) and median with interquartile range (IQR), respectively, while categorical variables were grouped into different categories. Descriptive statistics of variables were computed and presented in frequency tables or charts.

Ethical consideration

Ethical approval (ADM/DCST/HREC/APP/3572) was obtained from LUTH’s Human Research and Ethical Committee before conducting the study. The study was carried out in accordance with the Declaration of Helsinki (1964).

## Results

A total of one thousand, three hundred and forty-nine (1,349) cases of gynecological malignancies were managed at LUTH during the study period. During this period, VC accounted for 54 (4.0%) of all gynecological malignancies and an approximate average annual frequency of five cases per year. Out of the 54 cases of VC identified, only 45 (83.3%) cases had complete data and were included in the study analysis.

**Figure 1:**
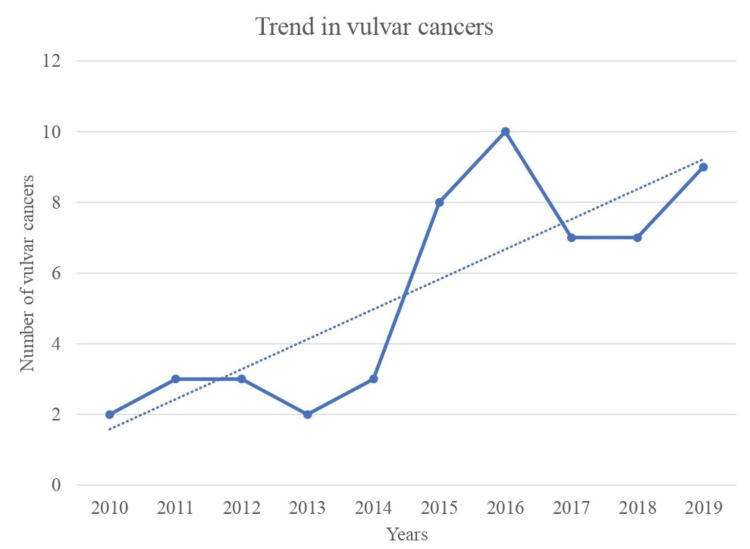
Trend in vulvar cancers over 10 years. There was an increasing trend in the number of vulvar cancers seen during the study period.

Figure 1 shows the trend in the number of VCs seen over the 10-year study period. There was a rising trend in the number of VCs managed within the institution, from two cases in 2010 to nine cases in 2019, with a peak of 10 cases in 2016. More than three-quarters, 41 (75.9%), of VC cases were managed within the last five years (2015-2019), compared to just 13 (24.1%) managed within the first five years (2010-2014) of the study period.

Table 1 shows the sociodemographic characteristics of women with VC. The majority, 27 (60.0%) of the women were above the age of 50 years at presentation with a mean ± SD age of 52.2 ± 11.8 years (25 - 74 years). Most of the women were married, 22 (48.9%), Christians, 36 (80.0%), of Igbo ethnicity, 20 (44.4%) with semi-skilled and unskilled occupations, 35 (77.8%), and had at least secondary school education, 26 (57.8%).

**Table 1:** Socio-demographic characteristics of women with vulvar cancers. *Others include Ijaw, Ibibio, Kanuri, and Tiv. n: total population; SD: standard deviation. The data has been represented as frequency (n), percentage (%), and mean ± SD.

VARIABLE	FREQUENCY (n=45)	PERCENTAGE
Age at presentation (Years)		
<40	9	20.0
40 to 49	9	20.0
50 to 59	15	33.3
≥60	12	26.7
Mean ± SD	52.2 ± 11.8	
Marital status		
Single	4	8.9
Married	22	48.9
Divorced	1	2.2
Separated	2	4.4
Widow	16	35.6
Educational status		
Primary	9	20.0
Secondary	26	57.8
Tertiary	10	22.2
Ethnic		
Yoruba	19	42.2
Igbo	20	44.4
*Others	6	13.4
Religion		
Christianity	36	80.0
Islam	9	20.0
Occupation		
Skilled	1	2.2
Semi-skilled	18	40.0
Unskilled	17	37.8
Unemployed	9	20.0

Table 2 shows the reproductive and sexual characteristics and risk factors for VC among the study participants. A large proportion of women, 19 (42.2%), has had at least five deliveries with a median (IQR) parity of 4 (1-6), while 18 (40.0%) had between two and four children with a median (IQR) of 3 (1-5). Most of the women, 29 (64.4%), had a history of multiple sexual partners, with the majority, 22 (48.9%), having two to three sexual partners, and the median (IQR) number of sexual partners was 2 (1-3). The mean ± SD age at coitarche was 19.9 ± 2.5 years; many women, 26 (57.8%), initiated sexual activities between 18 and 21 years, and more than half, 26 (57.8%), were postmenopausal.

**Table 2:** Reproductive and sexual characteristics and risk factors for vulvar cancer among participants. n: total population; IQR: interquartile range; SD: standard deviation. The data has been represented as frequency (n), percentage (%), median (IQR), and mean ± SD.

VARIABLE	FREQUENCY (n=45)	PERCENTAGE (%)
Children Alive		
<2	14	31.1
2-4	18	40.0
≥5	13	28.9
Median (IQR)	3 (1-5)	
Parity		
0	5	11.1
1 to 4	21	46.7
≥5	19	42.2
Median (IQR)	4 (1-6)	
Menarche (years)		
<12	1	2.2
12 to 14	24	53.4
>14	20	44.4
Mean ± SD	14.4 ± 1.3	
Menopausal status		
Menopausal	26	57.8
Pre-Menopausal	19	42.2
Multiple sexual partners		
No	16	35.6
Yes	29	64.4
Age at coitarche		
>18	7	15.6
18 to 21	26	57.8
>21	12	26.6
Mean ± SD	19.9 ± 2.5	
Number of sexual partners		
1	16	35.6
2 to 3	22	48.9
>3	7	15.6
Median (IQR)	2 (1-3)	

Most of the women with VC had a history of genital warts, 35 (77.8%), and vulva skin lesions, 40 (88.9%), before the diagnosis of VC. Only five (11.1%) were HIV positive, while two (4.4%) had a history of cervical dysplasia or cancer. Only a minority, 11 (24.4%) and two (4.4%) had a history of chronic use of steroids and smoking of tobacco, respectively (Table 3).

**Table 3:** Clinical risk factors for vulvar cancers among participants. n: total population The data has been represented as frequency (n) and percentage (%).

VARIABLE	FREQUENCY (n=45)	PERCENTAGE (%)
HIV infection		
Yes	5	11.1
No	40	88.9
History of genital warts		
Yes	35	77.8
No	10	22.2
History of cervical dysplasia or cancer		
Yes	2	4.4
No	43	95.6
Vulva skin lesion		
Yes	40	88.9
No	5	11.1
History of Smoking		
Yes	2	4.4
No	43	95.6
Chronic use of steroids		
Yes	11	24.4
No	34	75.6

The most common presenting symptoms of VC were vulvar swelling (32 cases, 71.1%), vulvar ulcer (15 cases, 33.3%), vaginal discharge (15 cases, 33.3%), and pruritus vulvae (14 cases, 31.1%). Similarly, the common examination findings in women with VC were vulvar mass (39 cases, 86.7%) and vulvar ulceration (nine cases, 20.0%) as shown in Table 4.

**Table 4:** Symptoms and examination findings in women with vulva cancers. n: total population. The data has been represented as frequency (n) and percentage (%).

VARIABLE	FREQUENCY (n=45)	PERCENTAGE (%)
Symptoms		
Asymptomatic	8	17.8
Vulvar swelling	32	71.1
Pruritus vulvae	14	31.1
Abnormal vulvo-vaginal bleeding	10	22.2
Abnormal vaginal discharge	15	33.3
Vulva ulcer	15	33.3
Vulva pain	2	4.4
Examination findings		
Vulvar mass	39	86.7
Vulvar bleeding	3	6.7
Vulva ulceration	9	20.0
Discharge	8	17.8
Abnormal skin lesion	8	17.8

**Figure 2:**
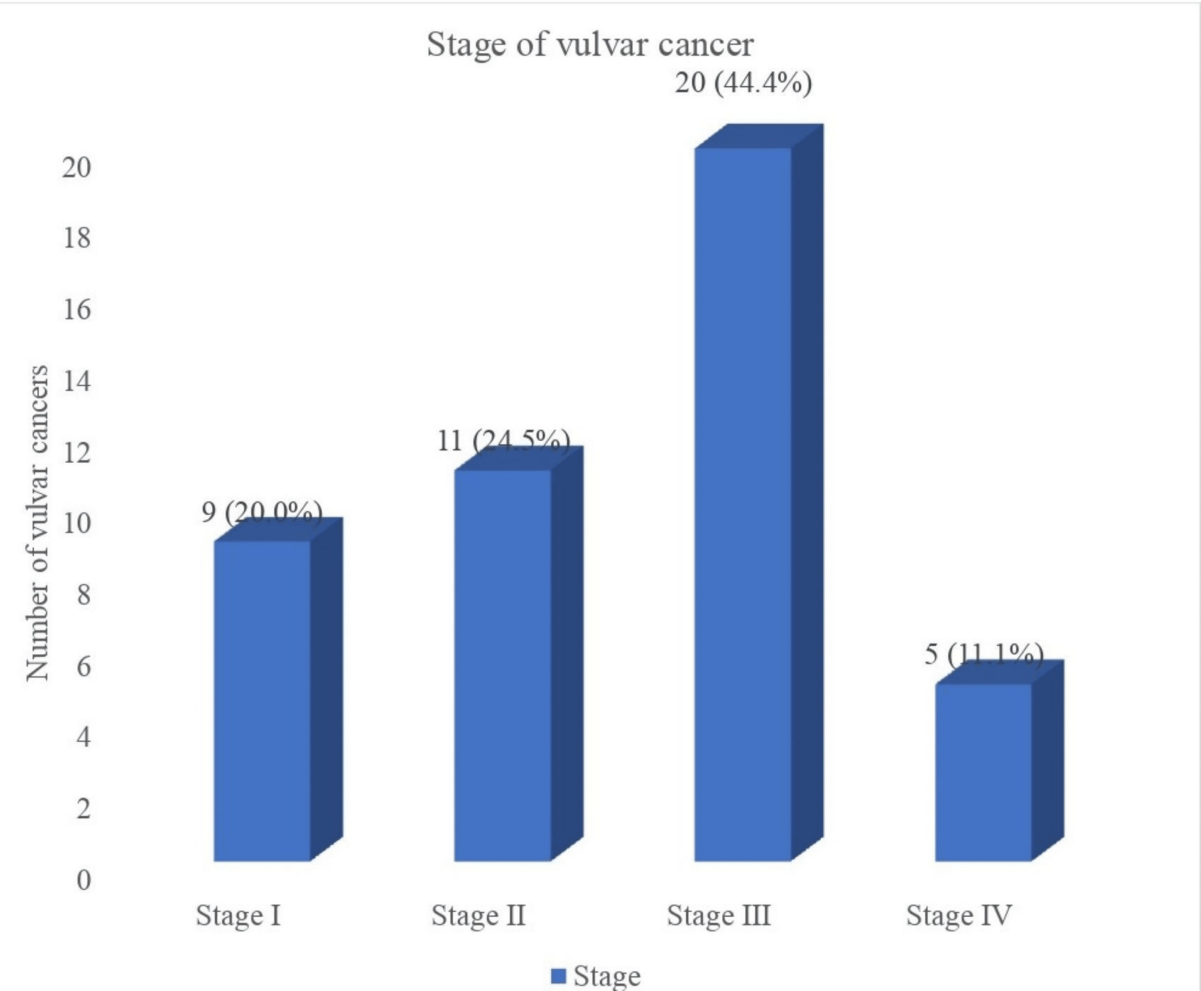
The stages of vulvar cancer at presentation. Most women with vulvar cancer present with stage III disease, followed by stage II, I, and IV disease.

Figure 2 illustrates the stages of VC. The majority, 20 (44.4%), of women with VC presented in stage III disease, while 11 (24.5%) and 9 (20.0%) presented in stages II and I, respectively. More than half of the women, 25 (55.5%), presented with late-stage disease.

**Figure 3:**
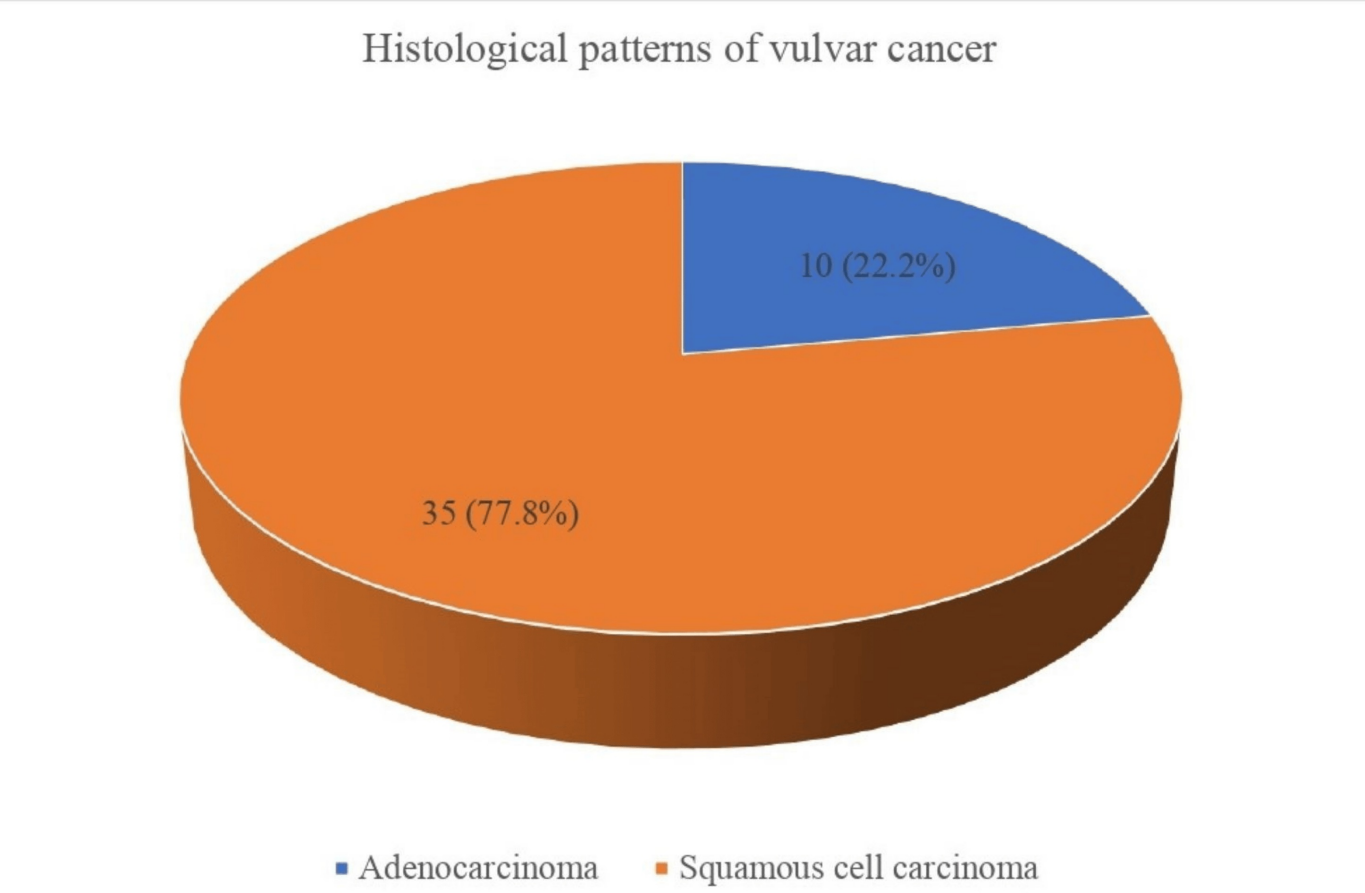
Histological patterns of vulvar cancer over 10 years. The most common histological type of VC was squamous cell carcinoma, followed by adenocarcinoma.

The most common histological type of VC among women in the study was squamous cell carcinoma, accounting for 35 (77.8%), while 10 (22.2%) women have adenocarcinoma (Figure 3).

Table 5 shows the various treatment modalities for VC during the study period. Surgery was the most common single modality of treatment for VC, 15 (33.3%), followed by chemotherapy, four (8.9%), and radiotherapy, four (8.9%). Chemoradiation, four (8.9%); surgery followed by chemotherapy, three (6.7%); and surgery and radiotherapy, two (4.4%), were the most common combination therapies received for the treatment of VC, while 12 (26.7%) did not receive any treatment, mainly due to financial inability to afford care.

**Table 5:** Treatment modalities of women with vulvar cancers. n: total population. The data has been represented as frequency (n) and percentage (%).

VARIABLE	FREQUENCY (n=45)	PERCENTAGE
Treatment modalities		
Surgery alone	15	33.3
Chemotherapy alone	4	8.9
Radiotherapy alone	4	8.9
Chemoradiation alone	4	8.9
Surgery and chemotherapy	3	6.7
Surgery and radiotherapy	2	4.4
Surgery and chemoradiation	1	2.2
No treatment received	12	26.7

## Discussion

VC is a rare disease that has not received much attention worldwide due to the investment of resources and focus of research on “the big three” gynecological cancers, i.e., cervical, ovarian, and endometrial cancers, which are more prevalent and associated with more cancer-related morbidity and mortality. Africa and other LMICs have contributed very little to the available epidemiological knowledge on VC, with minimal research output on the subject matter, leaving a knowledge gap in the understanding of the disease in the region. To bridge this gap, our study examined the trend in VC cancer over a 10-year period and described the epidemiological and clinical characteristics of the disease in Lagos State, Southwestern Nigeria.

VC accounted for 54 (4.0%) of the total gynecological cancers seen during the 10-year survey. This is in keeping with the widely reported 4.0-5.0% prevalence of the disease among other gynecological cancers [1,2,9,11,12]. Though this prevalence was similar to the 3.6% reported at Port Harcourt, South-southern Nigeria [15], it was higher than what was reported at Nnewi, Southeastern Nigeria (1.27%) [13], Zaria, Northwestern Nigeria (2.6%) [16], and Ibadan, Southwestern Nigeria (1.6%) [18], but lower than what was reported at Benin, South-southern Nigeria (5.7%) [14]. In Africa, VC accounted for 2.0% of all gynecological cancers in Ghana [19], while in Burkina Faso it accounted for 5.3% [20]. The variation in the prevalence of VC may be attributed to the variation in the prevalence of its etiological and risk factors, such as chronic vulvar dystrophies, HPV-related infections, and HIV/AIDS, in different populations. We found a rising trend in the number of cases of VC presenting to the institution for care during the study period. This is similar to the increasing incidence reported in the United Kingdom [21] and the United States of America [22] but contrary to the stable trend observed in Croatia [6]. Globally, a rising trend has been observed among elderly women, while a mixed trend was observed among young women [23]. This is mainly due to the increase in the aging population and the high prevalence of HPV infections [21].

In our study, the majority, 17 (60.0%), of the women with VC were within the menopausal age of 50 years and beyond, with a mean age at presentation of 52.2 years, and were menopausal (57.8%), in keeping with the assertion that VC is a disease of menopause, and that age plays a critical role in its epidemiology [21]. Compared to other centers in Nigeria, a lower mean age at presentation was reported in Ibadan (49.7 years) [18], Zaria (45.4 years) [16], and Port Harcourt (49.0 years) [15], while a higher mean age at presentation was seen at Nnewi (61.2 years) [13]. In Africa, higher mean ages at diagnosis were observed in Ghana (56.3 years) [19], Tunisia (65.4 years) [24], and Burkina Faso (55 years) [20], while a much higher age at diagnosis, between 70 and 73 years, was reported in high-income countries [6,12,25].

Approximately 80.0% and almost all (97.8%) of the women with VC in our study had secondary or primary education levels and low-level occupations, respectively, which suggests a low socioeconomic status (SES). This is consistent with findings by Ouh et al. [26], where VC was more prevalent in women with low SES. Low SES has been shown to be associated with an increased risk of developing HPV-related cancers due to more frequent exposure to sexual activities, which increases the risk of acquisition and persistence of high-risk HPV infections, and the poor uptake of HPV preventive practices by this group of women [26,27]. HPV infection is responsible for 40% of VC [11], and the rising rate of the infection in many LMICs has been implicated to be responsible for the high incidence of the disease in the region [10]. It is therefore not surprising that the majority of women with VC in our study had a history of multiple sexual partners, with approximately two-thirds having two or more sexual partners. Similarly, there was a high rate of prior genital wart disease among these women but a low rate of cervical dysplasia or cancer. This may be due to a low cervical screening rate, which is frequent among low SES and women with VC [26]. Vulvar dystrophies and vulvar intraepithelial neoplasm are common risk factors for VC, and these usually present with abnormal vulvar skin lesions, which were reported by the majority of women with VC in our study. Unfortunately, the actual diagnoses of these vulvar skin lesions are not known, as many women believe that they are signs of vulvar infections. As a result, they do not present early with these symptoms for proper evaluation but usually rely on self-treatment and over-the-counter medications only to present late when the disease has progressed. The prevalence of other known risk factors such as HIV/AIDS, smoking, and chronic use of steroids was, however, low among women in our study.

Vulvar swelling or mass, vulvar ulcer, abnormal discharge, and pruritus vulvae were the frequent presenting symptoms of VC in our study, which are consistent with the common symptoms reported in several studies [11,13,15,19,28,29]. Vulvar swelling or mass was the most common presenting symptom of VC in Lagos, similar to what was reported in other studies [11,19,21,28,30], while pruritus vulvae [13,24,29], vulvar ulcer [20,21], vulvar pain [15,20], and discharge [15] were the most common symptoms reported by other authors.

The majority (55.5%) of women with VC in Lagos presented with advanced VC. This is in line with findings from other regions in the country, with Zaria, Northwestern Nigeria [16], Port Harcourt, South-southern Nigeria [15], and Nnewi, Southeastern Nigeria [14], having between 80.0% and 100.0% rates of advanced-stage disease. A similar finding of late-stage presentation was observed in other African countries [19,24] and Iran [28]. On the contrary, the incidence of late-stage presentation of VC is lower in high-income countries, with rates ranging between 19.0% and 39.0% [12,25,30]. The most common stage at presentation of VC in Lagos was stage III disease. This is consistent with the stage at presentation in other regions in Nigeria [13-16] and Africa [24]. On the other hand, stage I disease is the most common stage at presentation in many high-income countries [12,25,30].

Squamous cell carcinoma was the most common histological type of VC in Lagos, accounting for approximately 80.0% of all the VC, which is in keeping with the findings in the literature [1], while adenocarcinoma accounted for the remaining percentage. The most common single treatment modality for VC was surgery alone in one-third of VC cases, followed by chemotherapy and radiotherapy alone, while chemoradiation and surgery followed by either chemotherapy or radiotherapy were the most common combination therapies. The use of radiotherapy and chemotherapy is a common and important form of treatment modality for VC in our environment due to the frequent presentation of advanced-stage diseases that are non-resectable, incompletely resected with positive surgical margins, or with nodal involvement. Unfortunately, more than a quarter of women with VC did not receive any treatment due to their inability to afford care. The majority of these women had advanced VC and could not afford the cost of chemo-radiation due to the lack of health insurance and government support for cancer care.

Limitations

The study was a single institutional-based study with a limited study population, which may not represent the true population characteristics of VC in the general population. As a result, these findings cannot be generalized. Being a retrospective study, its findings are limited by the availability, completeness, and quality of the data in the medical record. Furthermore, the use of paper-based medical records may also affect the quality of data collected. The small sample size also limits the accuracy and reliability of the study findings. A large prospective multicenter study is recommended for a more comprehensive and reliable evaluation of the epidemiology of VC in Lagos and Nigeria at large.

A major strength of our study is that it is the first study, to the best of our knowledge, to describe the trend, epidemiology, and clinical characteristics of VC in Lagos, Nigeria. This will serve as a baseline study for future epidemiological research on VC in the state and region.

## Conclusions

VC is a rare gynecological cancer in Lagos, Nigeria, accounting for 4.0% of gynecological cancers in LUTH. It is more prevalent among women with advanced age, multiple sexual partners, low levels of occupation, and postmenopausal women. The most common symptom at presentation is vulvar swelling or mass, and women with VC frequently present in advanced-stage disease, commonly stage III. The most common histological type is squamous cell carcinoma, and the majority of the women received either surgical treatment, chemotherapy, or radiotherapy, with a significant proportion of the women not receiving any care due to financial constraints. There is a need to increase public awareness about the disease among women to enhance early presentation, provide and subsidize preventive measures such as HPV vaccination, and treat women with VC to improve health outcomes.
